# Psychedelics as Tools for Belief Transmission. Set, Setting, Suggestibility, and Persuasion in the Ritual Use of Hallucinogens

**DOI:** 10.3389/fpsyg.2021.730031

**Published:** 2021-11-23

**Authors:** David Dupuis

**Affiliations:** Research Department, Quai Branly Museum, Paris, France

**Keywords:** psychedelics, cults and new religious movements, brainwashing, belief, ayahuasca

## Abstract

The use of psychedelics in the collective rituals of numerous indigenous groups suggests that these substances are powerful catalysts of social affiliation, enculturation, and belief transmission. This feature has recently been highlighted as part of the renewed interest in psychedelics in Euro-American societies, and seen as a previously underestimated vector of their therapeutic properties. The property of psychedelics to increase feelings of collective belonging and transmission of specific cultural values or beliefs raise, however, complex ethical questions in the context of the globalization of these substances. In the past decades, this property has been perceived as problematic by anticult movements and public authorities of some European countries, claiming that these substances could be used for “mental manipulation.” Despite the fact that this notion has been widely criticized by the scientific community, alternative perspectives on how psychedelic experience supports enculturation and social affiliation have been yet little explored. Beyond the political issues that underlie it, the re-emergence of the concept of “psychedelic brainwashing” can then be read as the consequence of the fact that the dynamic through which psychedelic experience supports persuasion is still poorly understood. Beyond the unscientific and politically controversed notion of brainwashing, how to think the role of psychedelics in the dynamics of transmission of belief and its ethical stakes? Drawing on data collected in a shamanic center in the Peruvian Amazon, this article addresses this question through an ethnographic case-study. Proposing the state of hypersuggestibility induced by psychedelics as the main factor making the substances powerful tools for belief transmission, I show that it is also paradoxically in its capacity to produce doubt, ambivalence, and reflexivity that psychedelics support enculturation. I argue that, far from the brainwashing model, this dynamic is giving a central place to the agency of the recipient, showing that it is ultimately on the recipient’s efforts to test the object of belief through an experiential verification process that the dynamic of psychedelic enculturation relies on. Finally, I explore the permanence and the conditions of sustainability of the social affiliation emerging from these practices and outline the ethical stakes of these observations.

## Introduction

The use of psychedelics to build or reinforce social connectedness and cohesion has long been observed by anthropologists (Harner, 1973; Furst, 1976; Dobkin de Rios, 1984). Among indigenous groups of the Western Amazon, ayahuasca^1^ is for instance mobilized during shamanic initiation or rites of passage to enable participants to have culturally prescribed experiences, such as encounters with culturally postulated supernatural beings (Harner, 1972; Reichel-Dolmatoff, 1972; Kensinger, 1973; Descola, 1996; Déléage, 2009). The properties of so-called “hallucinogenic” or “psychedelic” substances^2^ indeed make them strong supports of social affiliation. As they are deeply influenced by cultural background and social interactions, psychedelic experiences are powerful vectors of cultural transmission and consolidation of social groups (Dupuis, 2021).

The use of psychedelics in the initiatic rites of passage (van Gennep, 1960) and the coming-of-age ceremonies of many indigenous groups across the globe^3^ suggests that these substances are powerful catalysts and facilitators for enculturation and belief transmission. An important function of collective rites lies in their ability to induce a synchronization of affects and behaviors among participants, which has been identified as a powerful catalyst for social affiliation (Hove and Risen, 2009; Reddish et al., 2013; Tarr et al., 2014). Recent study (Kettner et al., 2021) suggests that psychedelics supports the “collective effervescence” (Durkheim, 1912) dynamic generated by rituals, leading to a “fusion of identity” and resulting in a sense of unity within the social group (Swann et al., 2009; Páez et al., 2015) that Victor Turner (1969) called “communitas”^4^. Drawing on cross-cultural data analysis, Grob and Dobkin de Rios (1992) and Dobkin de Rios et al. (2002) noticed that in contrast to Euro-American societies, where drug abuse is an important public health problem, especially among the adolescent population, many indigenous societies use hallucinogens in specific cultural settings – rituals – in order to enculturate youth. These authors suggest that psychedelics are in that context powerful tools in order to inculcate cultural values, beliefs or social norms, and thus to reinforce a sense of cultural identity.

The findings of the comparative ethnographic investigation that I am conducting for about 10 years within various shamanic centers of Peruvian Amazon (Dupuis, 2016) are congruent with this assumption. My ethnographic observations show indeed that the cultural elements as well as the features of the ritual devices specific to these institutions strongly influence the formal characteristics features of visual and auditory imagery (i.e., “hallucinations,” referred to in this context as “voices” and “visions”) perceived by the participants during psychedelic rituals.

The psychedelic experience is then often experienced by the participants as the concrete and tangible verification of elements of the local cosmological, etiological, or therapeutic knowledge. This knowledge is indeed embodied in the encounters with culturally postulated supernatural entities, occurring in a privileged way through “voices” and “visions.” Analyzing the social learning that underlies the emergence of these culturally prescribed experiences, I have called “socialization of hallucinations” (Dupuis, 2021) the dynamic through which verbal exchanges and social interactions shape the psychedelic experience by directing attention, expectations, and perception.

While observed for a long time by anthropologists, the property of psychedelics to facilitate belief transmission, enculturation and social affiliation has recently been highlighted as part of the renewed interest in psychedelics in Euro-American societies. The ability of these substances to support the emergence of “communitas” has for instance recently been seen as a previously underestimated vector of their therapeutic properties (Kettner et al., 2021). The property of psychedelics to increase feelings of collective belonging needs, however, to be considered carefully, as it raises complex ethical questions in the context of the globalization of these substances (Timmermann et al., 2020).

In the past decades, this property of psychedelics has been perceived as highly problematic by anticult movements and public authorities of some European countries. The emergence of “shamanic tourism” and the success of ritual practices involving the use of hallucinogenic substances have for instance given rise to serious concerns among the French public authorities, who have seen this as a potential source of “cultish driftings.” These concerns were expressed in particular by legal proceedings involving French members of Santo Daime and the “Takiwasi affair,” which led one of the main shamanic centers in the Peruvian Amazon and its French collaborators to be prosecuted by judicial authorities in France and accused of being a “cult group” (Bourgogne, 2011). In the course of this affair, the French public agency responsible for observing and analyzing the phenomenon of so-called “cultish” groups (MIVILUDES)^5^ and some anti-cult associations presented hallucinogens as “powerful drugs” implying “risks of manipulation” (MIVILUDES, 2005). The claim that psychedelics could be used for “mental manipulation,” psychological destabilization, unconscious or not fully consented conversion by cultish groups through so-called “chemical submission” (Pépin and Duffort, 2004) finally led to the prohibition of ayahuasca in France in 2005. By associating psychedelics with the notions of “mental manipulation” and “brainwashing,” this affair has strongly contributed to the social stigmatization of psychedelics and to the legitimization of their prohibition in Europe. Even though the trial was eventually dismissed, this case have indeed had an international impact, influencing public policies in other European countries^6^.

The concept of “brainwashing” was a key element of this case. This notion (also called “mental manipulation,” “mind control” or “coercive persuasion”) was first used by the anticommunist journalist and undercover intelligence agent Hunter (1951) in his book (1951) to describe the techniques through which Chinese Communist Party was supposed to make political opponents and American armed forces prisoners cooperate during the Korean War. In the 1970s, this notion became a popular concept through which was analyzed conversion among emerging religious movements^7^, including those associated with the hippie phenomenon, which were called “cults” by their detractors (Young, 2012). Brainwashing quickly became the central doctrine of American anti-cult movement, which approached emerging religious movements as “cults,” i.e., deviant and dangerous pseudo-religious groups using unethical manipulative techniques to recruit members^8^.

The American psychologist Margaret Thaler Singer was a prominent proponent of the brainwashing theory applied to religious movements and one of the leading theoretician of the anti-cult movement. In numerous mainstream publications (Singer, 1979; Singer and Lalich, 1995/2003), she has defended a theory allegedly inspired by academic research on communist “thought reform” programs (Lifton, 1954, 1961; Schein, 1956). Her conception of brainwashing was adopted from behaviorism (Bitterman, 2006), which was the dominant psychological theory when this concept emerged. In that perspective, brainwashing is thought as a process of intense manipulation based on conditioning techniques considered as able to force people to convert against their wills by altering people’s mental outlook and free will. Even if these conditioning techniques or the underpinnings of their effectiveness have never been precisely described by Singer, this conception has received an important popular diffusion under the name of “robot theory.” This theory claims that “cultish” brainwashing techniques are able to inhibit the free will in making critical decisions of members, who are then thought of as a kind of automatons devoid of personal autonomy and agency. In that perspective, people actually remains a member of so-called cultish groups against their will, because the group’s control on the member’s mind is total. “Cult” members are thus allegedly not responsible social actors and may be coerced in order to restore their autonomy (Shupe et al., 2004). Despite its lack of scientific support, this approach has played an important role in court situations and has formed the basis of the controversed practice of “deprogramming” used from the 1970s onward by some American anti-cult movements^9^ (Young, 2012).

Since the 1980s, there has been an intense public, scientific and legal debate about the concepts of cult and brainwashing. In opposition to the American anti-cult movement claims, voices raised to point out the unscientific and subjective nature of these concepts and to denounce their use as a rationale for persecuting unpopular new religious movements and for violating the constitutional rights of their members (Wessinger, 2017). After years of debates, an academic consensus has emerged, that brainwashing was pseudoscience. The brainwashing hypothesis as defended by Singer has indeed never been published in scientific journals, and thus has not been available for scholarly evaluation and critique (Anthony, 1990). The claim that so-called “cultish groups” could deprive individuals of their free will in the absence of physical force or threats through “conditioning techniques” had been then discredited by numerous research works, which have shown that “brainwashing” was an inadequate model for understanding the dynamics of conversion in any religious movement (Melton, 1999). Scholars pointed out, among other things, that the brainwashing hypothesis was based on an inappropriate application of the theory of conditioned reflex to social relationships, and on a misunderstanding of academic work on the thought reform programs conducted in Chinese prison camps (Robbins and Anthony, 1980)^10^. That consensus was capped in the U. S. Federal Court decision in the case of U.S. v. Fishman^11^. It has been then considered that the use of such arguments as a justification of deprogramming and legal action was illegitimate (LeMoult, 1978). Singer has been finally prohibited from testifying as an expert in American courts, due to the lack of scientific validity of her theories^12^.

It is nowadays widely accepted by scholars that the brainwashing theory was promulgated by the CIA for the sole purpose of serving as an anti-communist propaganda tool during the Cold War (Anthony, 2008), and that this theory was later reappropriated by the nascent anti-cult movement in the United States, serving as justification for highly controversial measures (e.g., “deprogramming”) against some new religious movements (Robbins and Anthony, 1980). In the United States, as in most countries around the world, concepts such as brainwashing are now considered by the courts to be unnecessary to justify investigations and possible prosecutions of religious groups suspected of breaking the law, including through the use of physical violence and coercion. Even if since the late 1980s the academic community have thus shared an almost unanimous consensus that the brainwashing thesis proposed by Singer and her colleagues was without scientific merit, this thesis has deeply impacted the popular imagination, as shown by its diffusion in many works of fiction, such as *The Manchurian Candidate*^13^. The concept of brainwashing, reformulated as “mental manipulation,” gained new resonance with public authorities in Europe in the 1990s (Richardson and Introvigne, 2004). This can be explained by a number of reasons (Melton, 1999)^14^. Perhaps the most important of these is that since the 1990s, violent incidents in Europe by minority religious groups such as the Solar Temple (Lewis, 2006) have come to the attention of the general public and public authorities. Since then, legislators, urged on by proponents of the “mental manipulation” theory and anticult associations, have been asked to create new laws and government agencies aimed at observing minority religious communities and identifying “cultish driftings.” In France, which is distinguished by its deep-rooted secular tradition, this shift was embodied in the early 2000s by the creation of MIVILUDES and the passing of a new law condemning the “fraudulent abuse of a state of ignorance or weakness” and aiming to repress the practices of “mental manipulation” allegedly used by “sectarian groups”^15^. Since then, courts have been asked to bring in rulings based upon testimony of experts in the controversed “brainwashing” perspective.

The prohibition of ayahuasca in France, as the Takiwasi’s legal proceeding – which was one of the first applications of the new law aimed at responding to the concerns of anti-cult associations -, should be thus understood in the light of this contemporary reemergence of brainwashing theory in Europe and its political and legal implications. For the French government, supported by the reports of MIVILUDES (2005) and the positions of anti-cults associations, psychedelics would therefore constitute potential tools of “mental manipulation.” As explained to me during interviews conducted between 2008 and 2013 with members of anti-cult associations and French governmental agencies, psychedelics are in that perspective considered as having the potential to reduce one’s agency and reflexivity and to impair one’s ability to make informed decision, allowing thus a fast and non-consented transmission of specific beliefs, i.e., to allow “brainwashing.”

Despite the fact that the capacity of psychedelics to produce “brainwashing” had already been explored unsuccessfully from the second world war onward^16^, these events bring back to the public debate the question of the place of hallucinogens in religious conversion and of their use as a potential tool for “mental manipulation.” If this can make one wonder about the possible emergence of a new witch hunt stigmatizing psychedelic users in Europe, these elements are to be relativized with regard to the worldwide dynamic of growing interest for psychedelics by the scientific and medical community as well as the general public. This point, however, highlights the fact that, despite the criticism that the “brainwashing” model has received, alternative perspectives on how psychedelic experience supports enculturation and social affiliation have been yet little explored. Beyond the political issues that underlie it, the re-emergence of the concept of “psychedelic brainwashing” can then be read as the fact that the dynamic through which psychedelic experience supports persuasion and transmission of beliefs is still poorly understood. These questions and their ethical stakes are, however, bound to become increasingly relevant, as the use of psychedelic substances is rapidly achieving mainstream status, especially in the context of the emergence of commercial interest for psychedelic therapy (Nichols et al., 2017), the so-called psychedelic science ‘revival’ (Langlitz, 2012), and ritualistic uses in secular and neo-shamanic contexts (Labate and Cavnar, 2014; Labate et al., 2016). Beyond the unscientific and politically controversed notion of brainwashing, how can we think the role of psychedelics in the dynamics of persuasion and transmission of belief?

Based on data collected during an ethnographic fieldwork in Takiwasi, one of the main shamanic center in the Peruvian Amazon, I propose here to explore this question. To do so, I will use an analytical framework rooted in interactionism (Becker, 1963; Goffman, 2002), narrative approaches (Cain, 1991; Seligman, 2005) and cultural phenomenology (Csordas, 1999) dialoguing with anthropology of belief and social psychology. Drawing on a case-study of a young French man participating to Takiwasi’s seminar, I will show that the dynamic of belief transmission is supported by a remarkable property of psychedelics, that of producing a state of *hypersuggestibility*. This property is, however, far from depriving the subject of reflexivity and agentivity as proposed by the brainwashing model. We will indeed see that it is also in its capacity to produce doubt and ambivalence that psychedelics support adherence, cultural transmission and social affiliation. I will show that it is ultimately on the recipient’s efforts to test the object of the belief through an *experiential verification* process that the dynamic of psychedelic belief transmission rely on. The dynamics of belief transmission supported by psychedelics, in that it depends on the efforts of the recipient, is consequently subject to significant inter-individual variations. I finally explore the permanence and the conditions of sustainability of the social affiliation that emerge from this dynamic in order to better seize the ethical stakes of these observations.

## Materials and Methods

To explore the aforementioned points, I will draw on data collected during an 18-month ethnographic fieldwork divided into three research stays, between 2008 and 2013. By providing thick descriptions of intricate cultural factors and interactional contexts shaping the hallucinogenic experience, the ethnographic method offers valuable insights to answer the questions explored in this article. By allowing for both first-person data collection (through participative observation) and third-person data collection (through interviews and observation of daily interactions), this offer some other major advantages. The cross-referencing of these two types of data offers indeed highly detailed informations about the phenomenological features of the psychedelic experience and makes moreover possible to build robust hypothesis about the interactional dynamics that shape it^17^.

The ethnographic research study was conducted in the San Martín region (Upper-Amazon region of Peru), most of the time in Takiwasi. During the first 4-month stay in 2009, I focused on the treatment of addictions proposed by Takiwasi. I used different methods of data collection, among which the study of daily and ritual interactions through the observation of participants played a central role, along with semi-structured interviews and life stories of about ten drug-dependent patients and the main actors of the institution (ritual specialists and psychologists, etc.). The second 6-month stay was in 2011, during which I attended four “seminars” offered to foreign clients. In each of these seminars there were between 15 and 20 participants. I shared the participants’ daily and ritual activities, as well as the discussion groups. I also conducted individual interviews with around thirty participants, and they shared their life stories with me. The 8-month final visit was in 2013. On this occasion, I attended three more “seminars,” that included a total of 45 participants. I also conducted weekly interviews with two drug-addicted patients who were hospitalized for 6 months and, more occasionally, with other patients. Since then, I have conducted through interviews a long-term follow-up of some Takiwasi’s clients through interviews in Europe and in Latin America.

This research study was also complemented with a comparative study, aimed at becoming more acquainted with the specificities of the institution under study in relation to the shamanic practices of the metis or Lamista people of the region. To this end, I carried out several short field surveys in the San Martin region (San Roque de Cumbaza, Chazuta, etc.), accompanying in their daily and ritual activities metis and indigenous healers offering their services to a local people within their village or to a national and international clientele within shamanic centers.

This ethnographic research study complied with the proceedings related to “informed consent” and was conducted with voluntary participants who were aware of the study objectives and knew that they could leave the investigation if they so desired. In order to protect the anonymity of the informants, all the names of the clients of Takiwasi have been replaced by pseudonyms.

### Context of the Study: The “Seminars” of Takiwasi, a Shamanic Center in the Peruvian Amazon

Participation in exotic rituals perceived as “traditional” and invested as therapeutic, religious or personal development practices have been increasingly popular with the Western public since the second half of the twentieth century. Fueled by the craze for the psychedelic brew ayahuasca as well as by the mythified image of the so-called primary forest, an influx of travellers has headed toward the Peruvian Amazon from the 1990s onward (Labate and Cavnar, 2014; Labate et al., 2016). The development of these practices has recently been analyzed by anthropologists as the emergence of a “shamanic,” “ethnomedical,” “mystical,” or “spiritual” tourism (Fotiou, 2010). Many reception centers for this clientele have appeared on the edge of the region’s metropolitan areas. These shamanic centers, most often based on the partnership between Westerners and Metis or indigenous people, offer an international clientele the opportunity to participate in ritual activities inspired more or less freely by the practices of the Peruvian metis shamanism (*curanderismo*), such as the ritualized use of ayahuasca.

Founded in 1992 by French physician Jacques Mabit as well as Peruvian and Spanish collaborators, the therapeutic community of Takiwasi is both an addiction treatment clinic and one of the most famous places in the region hosting Western travelers to “meet ayahuasca.” The premises are located on the outskirts of the city of Tarapoto, capital of the San Martin region, on a two-hectare plot of land bordered by a green fence and the Shilcayo River. In addition to a central building, which includes administrative and reception areas, an auditorium and a library, there is housing for resident drug addicts, a kitchen, two *malocas*^18^ where rituals are held, various workshops (carpentry, baking), a chapel, a laboratory for the production of phytotherapeutic products, a store and a botanical garden where the main medicinal plants used at Takiwasi are grown. The association also owns a 54-hectare plot of forest located a few kilometers away, in the *Cordillera Escalera* nature reserve, where there are 15 isolation huts (*tambos*) used for retreats (*dietas*), as well as a *maloca* inside which ayahuasca rituals are performed. Jacques Mabit is now the main authority in Takiwasi: the French doctor is indeed the last of Takiwasi’s founding members to still hold responsibilities within the institution. Since then, he has been the bearer of ritual authority, and all important decisions are subject to his approval. Apart from the Métis and indigenous healers (*curanderos*) sometimes recruited by the institution, only several people regularly officiate during the rituals. More recently, a priest has joined the team: he provides spiritual direction services as well as a weekly mass in the Takiwasi chapel, and regularly participates in the institution’s ritual practices.

Since its creation, the institution has developed a therapeutic device characterized by the reappropriation of elements of the indigenous pharmacopeia, such as emetic plants or ayahuasca. A team of doctors, psychologists and ritual specialists offers psychological support, medical follow-up and ritualized practices inspired by the Amazonian tradition, combining ritual and discursive elements from the region’s indigenous and metis shamanism, Catholicism and the New Age. These services are proposed in three different modalities: addiction treatment, which involves a 9-month internment, outpatient treatment and “personal development seminars.”

Seminar participants, both men and women, between the ages of 20 and 60, come from the middle and upper classes in the urban areas of French-speaking Europe and Latin America. They most often report a life journey marked by the accumulation and repetition of different registers of misfortune (grief, chronic pain or pathology, accidents, academic or professional difficulties, “loss of meaning”), the resolution of which is presented as the main reason for their coming. The resistance of these difficulties to the treatments offered by medicine and the dominant forms of psychotherapy (psychoanalysis, CBT, etc.) has most often initiated a process of experimentation with alternative therapies, of which the stay in the Amazon is a step. The coming to Takiwasi also reflects a form of religiosity characteristic of Western modernity (Luhrmann, 1991) based on the accumulation of “spiritual experiences” from various cultural backgrounds as well as on a modular, individual and irregular practice (Hervieu-Léger, 1999).

## Results: Psychedelics as Tools for Belief Transmission, an Ethnographic Case-Study

### The Psychedelic Ritual in Takiwasi

Bringing together about fifteen participants for a 2-week period, the personal development seminars offered by Takiwasi include various practices inspired by the metis Peruvian shamanism such as rituals focused on the ingestion of emetic plants and ayahuasca, and a few days retreat in the jungle involving the consumption of other vegetal preparations (*dieta*). Participation in these activities, accompanied by introductory lectures, integration sessions including speech groups and individual interviews, requires compliance with various food (pork, spicy condiments, alcohol), relationship and sexual prohibitions.

The first ayahuasca ritual takes place on Wednesday, the third day of the seminar, after the group’s participation in a purging ritual and several conferences. During these conferences, Jacques Mabit presented the program of the seminar as well as some elements of his etiological theory, including contamination by invisible pathogens of a “spiritual” nature. The experience of ayahuasca is then described as enabling the purification of the participant and his encounter with the “spiritual world” and usually invisible beings. Following an approach inspired by the work of some pioneers of the psychotherapeutic use of psychedelic substances (Grof, 1980), the ayahuasca experience is also presented as a psychological catalyst aimed at facilitating insights, emotional catharsis, childhood regression and understanding the tendencies and dispositions that organize the daily behavior of the participant.

Shortly after nightfall, participants are invited to join the main *maloca*, where about fifteen cushions are placed in a semi-circle. After immersing themselves in a purifying and protective bath of plants, participants take their seats around the ritual specialists. They have reserved places under icons representing Christ, the Virgin and Saint Michael, in front of which are placed their ritual tools (*mesa*). The ritual is frequently preceded by a reminder of the rules, such as body position requirements: participants have to sit, having to keep their back straight, to refrain from lying down and to abstain from sleep in order to “face what arises.” Participants are also invited to refrain from making noise, speaking, singing, interfering with, touching or talking to a neighbor. Ritual specialists usually justify these provisions by the fact that subtle elements are likely to be transmitted between participants through touch or sound. Participants are encouraged to “formulate an intention” before drinking ayahuasca, but not to focus on it during the ritual. In case of difficulty, they are allowed to call upon the officiants. Buckets are available if they feel the need to vomit, while forcing vomiting is not recommended. In case of emergency, it is possible to go to the toilets, located outside the *maloca*, but it will be necessary to report it and wait until a “cleansing” (*sopladas* of perfumes or tobacco) has been carried out by an officiant before returning to the ritual space. These rules will be in place until a ritual specialist turns the light on again, a gesture that marks the end of the ritual.

After these few words, a ritual specialist grabs a censer in which *palo santo*^19^ burns, goes around the *maloca*, then stops in front of each participant whom he invites into the smoke presented as purifying and protective. Jacques Mabit then makes a few gestures to materialize the limits of the space occupied by the participants, using holy water and salt, which he spreads behind and between them. These actions, presented to the participants as delimiting a “ritual circle” capable of protecting them from negative interactions, therefore imply compliance with the rules concerning their entry and exit. The master of ceremonies then sings several songs (*icaros*) and blows tobacco smoke (*mapacho*)^20^ – presented as protective – on his ritual tools, including the bottle containing ayahuasca. Everyone is then invited, one after the other, to come and have a cup of the drink. The light is then turned off. Several songs rise up, recited by the various officiants, while one of them walks by the participants, blowing tobacco smoke on the plexus, fontanel and hands (*soplada*) of each of them. After which come the recitation of an exorcism prayer in French, other songs, as well as a second series of sopladas, this time made with the help of *agua florida*^21^. The officiants’ songs, recited in Spanish, Quechua and French, will then follow one another until, 6 to 8 h later, the effects of the drink dissipate.

### The Infestation Diagnosis

In Takiwasi, the dynamic of enculturation and belief transmission is most often initiated by a “diagnosis of infestation” formulated by the ritual specialists to the participants during integration sessions. This diagnosis is based on the etiological and therapeutic theory that characterize Takiwasi and distinguish this institution from both the Peruvian *curanderismo* and other shamanic centers of the region. The central element of this theory is the concept of “infestation.” Borrowed from Catholic theology, this concept initially refers to a more minor and common mode of demonic influence than possession, characterized by the presence of a demonic entity abusing the subject by affecting his health, faith or thoughts (du Clos, 2007). In Takiwasi, infestation is thought of as a parasitic relationship with one or more malicious supernatural beings of a demonic nature. This condition, which is also presented as the cause of physical and psychological disorders, is thought of as the consequence of the transgression of taboos (drug use, sexuality, magic practices, spiritualism, etc.), contact with places or people, or transmission through relations of filiation. Finally, this condition is presented as requiring specific treatment, implying the purification of the patient through the absorption of emetic preparations, ayahuasca as well as practices proposed by the Catholic Church, such as exorcism (Dupuis, 2018a).

This theory, developed by the main actors of the institution over the past 20 years, highlights the increasing use of the Catholic doctrinal body and the ecclesial institution, which has progressively influenced the form and function of the practices proposed by Takiwasi (Dupuis, 2018b). The ayahuasca ritual is now characterized by the use of exorcism prayer, the crucifix, holy water, and the mobilization of the main figures of the Catholic pantheon. If the integration of elements of popular Catholicism is a classic component of the metis shamanism of Amazonia (Taussig, 1987; Gow, 1987; Chaumeil, 2003), the central place given here to the motif of demonic possession, the functions of exorcism and evangelization attributed to the use of ayahuasca, as well as the attachment of these practices to the ecclesiastical institution and to Catholic demonology are more original. The evolution of Takiwasi brings now the institution closer to the charismatic, evangelist, and Pentecostal movements which are widely spread in Latin America. In this sense, Takiwasi offers an exemplary case of contemporary cultural recompositions of so-called “shamanic” practices centered on the use of ayahuasca that are currently emerging in the Amazon (Labate and Cavnar, 2014; Labate et al., 2016).

The infestation diagnosis is usually realized during private interviews or speech group integration sessions. The day after the ayahuasca ritual, the participants are in fact invited to meet collectively in the early afternoon to participate in a discussion group called “post ayahuasca.” In the presence of Dr. Mabit and one or more of his assistants, participants are asked to narrate in turn and in front of everyone their experience of the ayahuasca. The ritual specialists then give some comments to the participant. Adrien, a young Frenchman in his early twenties who came to participate in the Takiwasi’s seminar in order to “discover traditional Amazonian medicine” and to “work on oneself,” is subject to a diagnosis of infestation during the first integration session of the seminar. During the speech group, Adrien presents his ritual experience of the day before as difficult to understand and marked by several unpleasant sensations:

“I was super cold at the first part, because my hair was wet. I was blocked, closed, I needed a first contact, a first confidence building. So I contacted the ayahuasca, we started things (.). I was afraid, it was very unpleasant (.) And at one point I thought I was going to suffocate too. I thought I was going to die. I was really, really thirsty.”

He also reports feeling a certain distrust of ritual specialists, expressed in particular in the discomfort he felt when he came into contact with a medal^22^ given by Jacques Mabit (“Jacques you gave me a medal, which I kept for a while. Then at a certain point I became saturated, so I put it down, it weighed me down, it haunted me, I had the impression that it was blocking me”) and which culminates in a vision assimilating the officiant to a demonic figure (“I was afraid of you in fact, because I saw you in a diabolical way at first”).

The account of Adrien’s ritual experience leads Jacques Mabit to diagnose the signs of a “presence” described as “parasitic.” This diagnosis is based on the sensations reported by the participant, presented by the ritual specialist as a symbolic language that should be deciphered. Jacques Mabit begins his comment evoking the sensation of cold felt by Adrien. This sensation is, at first, presented by the ritual specialist not as a normal perception of temperature, but as an element manifesting a daily psychological disposition of the participant, a tendency to “escape in front of fear.” Dr. Mabit then invites Adrien to look for the reason for the fear felt during the previous day’s ritual. The young man mentions his fear of the ritual specialists, to whom he attributed malicious intentions and whom he perceived “in a diabolical way.” This point is interpreted by the officiant as the manifestation of a disposition governing the participant’s daily life consisting of a “fear of being manipulated, programmed without your knowledge, that someone has a hold on you in some way.” Jacques Mabit also indicates that this fear, here “projected” onto the ritual specialists, would in fact find its origin in Adrien’s infantile interactions and kinship relationships. As the young man evokes “a fear of losing spiritual knowledge,” Jacques Mabit invites him to examine the nature of this “knowledge,” suggesting that it is the support of a “hold” of “spiritual connections” serving the intentions of a “manipulative spirit.” The sensation of cold, first interpreted as the manifestation of a psychological disposition, is then presented as a “spiritual cold” signaling a “parasitic presence.”

The presence of a “manipulative spirit” endowed with an intention and an agentivity of its own is thus posited, presented as a parasitic agent pursuing interests antagonistic to those of its host. This entity would manipulate Adrien by influencing his desires, thoughts and emotions without him being aware of it, leading him to confuse his own mental states with those induced by the parasitic entity. The presence of a parasitic entity would result in a loss of discernment of the participant as to the origin and nature of his own states. Thus, Jacques Mabit suggests that the fear reported by Adrien is in fact the fear felt by the entity when it comes into contact with the ritual work, which aims precisely at driving it away.

As we can see, some elements of Adrien’s account are interpreted by the officiant as the signs of the manipulative influence of the parasitic entity, revealing both the presence of the latter and its intention, consisting of opposition to the actions and intentions of ritual specialists. The intense thirst reported by Adrien, like the cold mentioned above, is indeed presented by the ritual specialist not as a normal perception but as a “suggestion” of the parasitic entity aiming at leading Adrien to leave the ritual space. It is thus understood that if the entity aims at expelling him from the ritual space, it is because of the effectiveness of the actions of the ritual specialists which aim at fighting against its presence. The ambivalent relationship reported by Adrien with the medal is also interpreted by Jacques Mabit as a sign of manipulation by the parasitic entity. Adrien’s ambivalence toward the medal would thus illustrate the participant’s lack of discernment in the face of the intentions of this entity, whose interest here is to keep the artifact away so as not to be discovered and expelled. Despite the discomfort felt, Mabit suggests consequently that Adrien should not abandon the object given by the ritual specialists.

As we can see, the diagnosis of infestation is based on an epistemic asymmetry that endows the ritual specialist with an epistemic authority. In the former sequence, Dr. Mabit has indeed positioned itself as having a capacity to “see” of which Adrien is deprived, and that allows him to identify the presence and nature of an invisible entity with which the young man is in relation without being conscious of it. Where Adrien seems not to have noticed this presence, Mabit claims to have fought against it during the ritual and seems to know it intimately, describing its intentions, its strategies and its weaknesses (“the little beast doesn’t like the medal”). Adrien’s diagnosis thus leads the officiant to deliver to the group of participants the typical reactions – fear, discouragement – that a discovered “parasitic entity” will try to arouse in its host in order to escape the operations of the ritual specialists.

However, Jacques Mabit does not claim to have a full understanding of the events of the previous night. To Adrien, who asks him “of what order” this “parasitic presence” is, he replies that he “does not know.” Dr. Mabit therefore invites the participant to investigate by himself during the next rituals on the nature and origin of this parasitic presence. The ritual specialist does, however, offer some etiological suggestions, such as “deep emotional wounds.” The officiant also suggests that certain practitioners of “energy techniques” that Adrien had frequented in previous years are likely to be “sorcerers,” which he defines as “a magician who uses energies, who works for the forces of evil.” These “sorcerers” are presented here as individuals with malevolent intentions and supernatural powers they derive from their association with demonic beings. Mabit thus poses as a possible origin of the infestation the frequentation of these evil magicians, who would be difficult to identify because of their “seductive” character. The ritual specialist finally mentions “bad and dangerous” techniques such as Reiki. Jacques Mabit ultimately suggests to Adrien, who seems doubtful about the proposed diagnosis, to “observe” during the next ayahuasca ritual in order to “look face-to-face” and verify the proposed diagnosis by himself. Faced with the attacks of the “parasitic presence,” Adrien is finally invited to “hold on” to the ritual specialists and the artifacts that they have given him.

### Doubts and Reflexivity: The Dynamic of Belief

The diagnosis of “infestation” formulated by the ritual specialists during integration sessions is far from giving rise to an automatic adherence. During our first interview conducted shortly after the speech group, Adrien seems to be very critical of the diagnosis of infestation proposed by Jacques Mabit which he attributed first of all to a “paranoia” as well as to mercantile intentions:

“The more you believe in demons, the more you attract them. The more you are in protection, the more you attract evil. (.) Finally, it’s only suppositions, I don’t know, a kind of paranoia (.) I’ve seen some people, who are specialists, they love demons, they always feel possessed, so they are necessarily possessed! (.) It’s also good to say “you’re possessed,” it means that there’s work to be done, money to be earned!”

In spite of these criticisms, the young man nevertheless evokes during our interview the possibility of being the object of an “infestation,” the existence of which he does not seem to contest. This partial acceptance of the diagnosis seems to be based on the fact that the latter have initiated an investigation into the origin of these “parasitic” entities:

“I have done quite a few rituals, I have worked on a spiritual level on things that I do not master. For example, I had courses with someone I did not know well, advanced courses of healing at the spiritual level. He did some rituals that I didn’t practice, but I taught them, and then I practiced them. And there I may have picked up some stuffs. Maybe in the Philippines with the healers, I don’t know.”

Adrien thus seems to occupy an ambivalent position, oscillating between doubt and adherence. If at first he seems very critical, we can see that he does not reject the existence of demonic entities or acts of witchcraft. He thus seems to oscillate in an undecidable way about the diagnosis that has been proposed to him, which is revealed by his joke at the beginning of our interview: “Yes, I’m ok to discuss with you a bit. I mean knowing that I am possessed. So what I am saying is a projection of my demons (laughs)!”

If some participants, like Adrien, affirm that they “do not believe” in the proposed diagnosis in the sense of a full and complete adherence, they nevertheless most often show an indeterminacy oscillating between doubt and adherence. Adrien’s account illustrates his reluctance to reject firmly the infestation diagnosis proposed by Dr. Mabit. It is quite striking that the object of this ambiguous adherence is designated by Adrien in a vague way (“some stuff”). This approximate evocation recalls the mysterious character of the proposals of the ritual specialists who mobilize signifiers such as “energy,” “energetic body,” “demon,” “parasitic entity” or “sorcerer” whose content never seems to be fully grasped by the participants. Because of this mysteriousness, the diagnosis proposed by the ritual specialists usually provokes an evocative process in the participant, who seeks to clarify its meaning.

The gradual generalization of the use of the categories proper to the local etiological-therapeutic theory is thus far from revealing the adoption of a univocal and complete meaning of this knowledge. The appropriation of the interpretative system is, as we can see, not free of doubts and criticisms. The participants, even when they criticize the proposed diagnosis, seem nevertheless unable to exclude the potentiality of its relevance. Adrien’s position thus illustrates that of most of the participants who, far from showing full and definitive adherence to the proposals of the ritual specialists, seem more compelled by their participation in the device to consider, at least temporarily, the possible relevance of these proposals.

### Believing, Betting, Committing

As Adrien’s reaction at the end of our interview reveals, this strategy is most often concretized by a search for verification of the proposition causing undecidability, in this case the infestation by parasitic entities diagnosed by the ritual specialist:

“I would have seen it, I would have believed him, I may see it, I don’t know (.)”

When the young man declares that he “does not believe,” he does not exclude the possibility that the diagnosis is true, but he says that he refuses to accept it without verifying it by “seeing” it, the experiential dimension being the decisive criterion for validating this verification. This search for *experiential verification* is presented by the ritual specialists as likely to be carried out through participation in the next rituals, described as capable of providing proof of their proposals. The ritual specialists thus frequently invite the participants to “verify for themselves in the ayahuasca” the knowledge which they expound during the discursive interactions. This invitation appears to be all the more attractive because the possibility of an experiential verification of the diagnosis is embodied in the figure of the ritual specialist, who presents the origin of his knowledge as strictly empirical.

As illustrated by this testimony from another seminar’s participant, the persistence of an illness that has resisted previous therapeutic proposals also invites many participants to adopt this etiological system on a temporary basis. They usually do so in order to test its effectiveness, since they are interested above all in the possible benefits of the cure:

“I don’t really know what to think about all this, about these stories of demons, of transgenerational (.). So I don’t know, but anyway, I can’t stay like that, so, well, I’ll play the game and we’ll see what the result is. Anyway, I have nothing to lose by trying!”

These calculations of interest often seem to take precedence over the question of the veracity of the proposals of the ritual specialists and lead most of the participants to follow their invitations and recommendations, even though they remain ambivalent about their initial premises. In spite of his doubts and after considering leaving the seminar, Adrien decides to participate in the following rituals in order to benefit from the therapeutic proposals based on the diagnosis of infestation:

“Apparently, the priest, he also saw that I was parasitized. That’s why they offer me after the mass, something to free me. Well, we’ll do that. (.) I’ll do the thing to the end.”

Thus, during the course of the survey, I saw many participants going to the Masses proposed during the seminar, even though they declared themselves to be atheists or were very critical of the proposals of the ritual specialists concerning the “demons.” This behavior, which illustrates in a striking way the paradoxical character of the dynamic of belief, seems to be based on a calculation of interest which is not without evoking a form of “Pascalian wager”:

“Yesterday I went to mass before the ayahuasca session. I am not really religious, at least not Christian, and besides, my family is originally Lutheran! But I don’t know, it was cool to be with everybody. And I guess I also thought that if all these stories are true, it’s better to be on the right side. And anyway, it’s not going to kill me to go to Mass!”

### The Experiential Verification of the Infestation Diagnosis

A few days after the first discussion group, the members of the group go to the plot of forest next to Tarapoto where the *dieta* will take place, a few days retreat in the jungle that will begin the next day. At nightfall, they join the *maloca* to participate in the second ayahuasca ritual. During an interview carried out on the return from the *dieta*, Adrien reports having during this ritual “expelled spirits” that he thinks he had contracted when taking psilocybe mushrooms a few years earlier. He describes this sequence as a “teaching” of ayahuasca and “animal spirits” perceived through visual hallucinations:

“There were spirits that came out. Faces that I had seen in a piece of wood while taking mushrooms [.]. I hadn’t noticed that it had actually come in. I was even amused by the faces I saw in the wood. But then it came out (during the ayahuasca ritual) and I realized that it was not good. I saw the same piece of wood with a head in it again, so I saw that I had caught it while taking the mushrooms. I saw it in vision and I threw it up, so I threw up air, I was burping, I was sighing. I also got the information that I shouldn’t do anything with the plants (psychotropic ones). And that’s the snakes, the ayahuasca and all the animal spirits that passed by, that came in, that taught me that.”

The presence of parasitic entities, the discovery of the contaminating nature of certain practices, the encounter with pedagogical and protective entities: on returning from the *dieta*, the sequences reported by the members of the group show a striking adequacy with the belief system exposed by the ritual specialists during the preceding discursive interactions.

During the last interview that he grants me at the end of the seminar, Adrien presents the 2 weeks spent in Takiwasi as an investigation initiated by the partial and mysterious diagnosis of infestation proposed by the ritual specialists during the first speech group. The origin of the infestation is finally attributed by Adrien to the “wild” use of psychotropic substances and to “magical” practices involving the opening of “tunnels” that the participant had never taken care to close. One recognizes here the main features of the local etiological theory: opening of the “energetic body” or the model of parasitism and contamination, which are here imputed to the realization of socio-moral infractions. Although he presents these elements as hypotheses, the young man nevertheless displays his determination not to reproduce the actions that he believes may have caused the infestation. Thus, during our conversation, he avoids reproducing in front of me the gesture that he considers as a potential vector of contamination, which consists in tracing with his fingers a pentacle supposed to “open energy tunnels.” At the end of the seminar, the young man presented himself as having finally understood and verified the diagnosis proposed by Jacques Mabit. The mistrust he had shown toward Jacques Mabit, Takiwasi and its ritual practices during our first exchange finally gave way to recognition, to such an extent that he invites me to “forget” what he had told me during our first interview, which he now interprets as “projections of his demons.”

## Discussion

### The Ambiguities of Psychedelic Enculturation: Doubts, Reflexivity, and Belief

This ethnographic case-study illustrates in an exemplary way the belief transmission dynamic based on psychedelic experience in Takiwasi. As illustrated by Adrien’s case, this experience is far from inducing a sudden, linear and automatic conversion. The psychedelic ritual seems rather lead participants to an oscillating indetermination between doubt and adherence, which strongly mobilizes the reflexivity and agentivity of the recipient.

Beside uncertainty, the kind of belief induced by the participation to psychedelic ritual is moreover characterized by the vagueness of the object of belief. The vague and elusive character of the signifiers composing the belief system transmitted by the specialists drapes them with an indeterminacy that allows them to cover varied and flexible – and sometimes even contradictory – interpretations. I could for instance observe during my investigation in Takiwasi a great variety of representations of “demons” among the participants. Some see them as a metaphor for emotional or behavioral complexes, while others show a more complete adherence to the propositions of ritual specialists concerning the existence of invisible entities. As illustrated by the testimony of this Peruvian drug addict patient at the end of treatment in Takiwasi, others still consider this possibility, while considering that it may also be, and at the same time, the product of projective mechanisms:

“The others (patients) talk about demons but I don’t think it influenced me much, they didn’t really talk to me about it. I must have seen demons eight or nine times in ayahuasca sessions. It started around the second or third month of treatment. Mostly they would watch me, hide and try to intimidate or seduce me. (.) I’m a bit dubious about the accounts of visions from some of the companions. Some of them seem to me to be telling themselves stories or projecting their stuff that they take too seriously. Jeremy has a digestive problem and, for him, it is an entity. I don’t believe him, it’s probably a bacteria or a parasite. Jeremy sees a lot of entities and demons, I’m more cautious about these things. I know that possession is possible, it exists. But honestly, I don’t have the experience or knowledge to discern, to know what is and isn’t.”

The majority of the participants balance irresolutely between the possibility that there are invisible demonic entities and the possibility that the “demon” is a metaphor for knowing themselves better or for personifying their suffering and misfortune. The signifiers that make up the belief system of the ritual specialists thus constitute plastic terms that are easily appropriated and to which an idiosyncratic and mobile meaning can easily be attributed. The vague and elusive nature of the signifiers composing the etiological knowledge transmitted by the local epistemic authorities is reminiscent of the “floating signifier” described by Claude Lévi-Strauss (1950). These signifiers designate notions – such as mana, a central concept in Melanesian rites, but whose meaning remains floating or vague – whose function “is to oppose the absence of meaning without having any particular significance in itself” (Lévi-Strauss, 1950). These signifiers thus have the advantage of allowing the experience to be socialized without requiring a strict consensus of the actors on the meaning of the experience. As this seminar participant testifies, their properties will eventually allow people to remain in this state of undecidability without feeling the need to make a definitive decision about the nature of his experience:

“I mean I don’t know, I can’t be sure, really know if they are entities or psychological projections. You never know. Sometimes, during ayahuasca sessions, I see an eagle and I have the impression that it is the spiritual protection of my grandmother, but in fact I don’t know. But still, when I see the demons it is very clear, they are not vague shadows or my imagination, I see them clearly.”

The appropriation of the local belief system supported by the participation to the psychedelic ritual is consequently composed of doubts, criticisms, and allows the maintenance of multiple – and even contradictory – interpretations about the meaning of the object of belief. Adherence to the interpretative framework proposed by the ritual specialists is in this sense reminiscent of the concepts of representational (or reflexive) beliefs proposed by Dan Sperber to describe the relationship of faithful to religious propositions (Sperber, 1982, 1990, 1996)^23^. Unlike factual (or intuitive) beliefs, which are more intuitive because they are attached to perceptual anchors that tend to make them unquestionable, the content of representational beliefs is not entirely grasped by those who hold them. Insofar as they are transmitted by authorities, they are nevertheless assumed to be true and preserved by means of “quotation marks.”

Integrating the enigmatic statements of the ritual specialists without fully understanding them, the seminar’s participants thus explore various possible interpretations in order to determine the one that will be the most relevant for them. This field of interpretation is relatively free: if the path is certainly marked out by the propositions of the local epistemic authorities and the normative constraints mentioned above, it remains, however, specific to each person. The participants, even when they criticize or don’t fully understand the local cosmological and etiological theories proposed by ritual specialists, seem nevertheless unable to exclude the potentiality of their relevance. They consequently usually try to verify them by participating in the following rituals.

We understand from Adrien’s account that his reluctance to firmly reject the interpretative system proposed by ritual specialists is based on doubts induced by the psychedelic ritual experience, some aspects of which are very likely to be interpreted as tangible verification of the proposed diagnosis. This dynamic is reminiscent of the negative mode of belief formulated by Severi (2015), who proposes to consider the mental state of belief more as “a dubious not knowing” based on an “incomplete constellation of clues” rather than a clear and positive adherence to a proposition. This ambiguous adherence characterizes in particular the participants who, as Adrien, present themselves as circumspects or opposed to the possibility of the existence of supernatural entities. The position they display evokes the dynamic proper to bewitchment diagnosis in contemporary French rural populations analyzed by Favret-Saada (1980). Borrowing this model from the ethnologist and psychoanalyst Mannoni (1969), she shows that the subjects of bewitchment diagnoses are caught in a contradictory tension summarized as follows: “*I know well* (that malevolent sorcerers do not exist)…*but still…* (some aspects of my experience seem to accord with the existence of such beings).” In a dynamic evoking this model, Adrien’s psychedelic ritual experience seems to be able to sow doubt, to undermine his certainties, thus leading him to consider the possibility of the relevance of the officiants’ diagnosis, even if it is based on propositions in which the participant affirms that he does not believe.

The specific mode of belief induced by psychedelics reminds in this regard the attitude of belief as described by French anthropologist Hamayon (2006), which “accommodates the expression of doubt (.) (and) the absence of certainty.” In line with this work and based on her ethnographic investigation on a Korean new religious movement, Luca (2013) has emphasized the instability of “belief” by defining it as “the oscillation between being and not being (.) the margin between the two,” which characterizes the position of many participants in the face of the proposals of ritual specialists.

The participation in the ritual induces in this sense a position of undecidability concerning the propositions of the ritual specialists, which evokes the situations of cognitive dissonance described by social psychology (Festinger, 1957; Harmon-Jones and Judson, 1999). In these situations, one simultaneously experiences contradictory elements of knowledge. This position then leads one to try and make these irreconciliable elements of knowledge concur in order to maintain a coherence and to reduce the psychological tension induced by undecidability. The psychedelic ritual appears then as inviting to question and *verify* in a concrete and tangible way the object of belief, and plays in this sense a central role in the participant’s subsequent involvement in ritual practices. As illustrated by Adrien’s journey, the doubts and uncertainty caused by the psychedelic experience are powerful vectors of commitment in the ritual practices. It is consequently paradoxically in their capacity to produce ambivalence and reflexivity that psychedelics support the belief transmission dynamic.

### Experiential Verification: The Underpinnings of Psychedelic Enculturation

The sensitive and bodily experience plays here a key role in the verification dynamic caused by the ambivalence experienced by the participation to the first steps of the seminar. Adrien’s case suggests that it is indeed because it is haloed by the evidence of the senses that the psychedelic experience is then leading Adrien to the adherence to the system of belief that surrounds it. The components of the perceptive experience, in that they present for those who experiment them a character of evidence difficult to question, constitute privileged vectors of adherence (Bem, 1970).

The central place given to the sensory experience and embodiment in the dynamics of the conversion and religious transmission, which appears prominently in the case of the hallucinogenic rites, has been observed by the ethnographic works in various cultural areas (Luhrmann, 1991, 2012; Csordas and Harwood, 1994; Huang, 2003; Mahmood, 2011; Kopenawa, 2013). Although it is thus not restricted to the religious movements proper to the Western modernity, it constitutes, however, one of the characteristic features of it, particularly salient in the new religious movements of New Age inspiration (Luhrmann, 1991). Their settings often surround the ritual practices with discursive sequences explaining the ontological, etiological and therapeutic belief system associated with these practices, while paradoxically underlining the importance of the individual’s sensory experience, which is here a decisive normative criterion (Houseman, 2012). The adherence to the belief system exposed during the discursive sequences is then supported by the bodily experience. The latter, charged with sensory and emotional intensity induced by the participation in the ritual practices, is frequently seen as a concrete and tangible verification of the local belief system.

This singular economy of adherence, which I called here *experiential verification*, characterizes a Western religious modernity marked by the growing importance of individual experience and the rejection of a strictly propositional adherence, which until then supported inherited religious identities (Hervieu-Léger, 1999). These observations contrast with the representational conceptions of religious transmission diffused by so-called cognitive anthropology in recent decades (Sperber, 1996; Boyer, 2001; Atran, 2005), which has reduced this dynamic to a set of automatic mental and propositional operations (Severi, 2004). The dynamic of enculturation supported by the psychedelic rituals draws our attention to the aspects neglected by this approach: the social interactions processes, their ecological factors and their experiential underpinnings (Dupuis, 2021).

### Psychedelic Rituals as Suggestibility Techniques

A remarkable property of psychedelics seems to play a central role in that experiential verification dynamic: their ability to increase suggestibility. This property, already observed by the first wave of research on these substances (Leary, 1961), has been further emphasized in contemporary research studies (Carhart-Harris et al., 2015; Timmermann et al., 2021). These works claim that, like hypnosis (Lynn et al., 2015), psychedelic substances produce a state of enhanced suggestibility^24^. Drawing on cross-cultural data analysis, Grob and Dobkin de Rios (1992); Dobkin de Rios and Stachalek (1999), and Dobkin de Rios et al. (2002) have suggested that hallucinogenic substances are used by many indigenous groups to create hypersuggestible states of consciousness, in order to promote fast-paced socialization for religious or pedagogical purposes, as enculturating adolescents during puberty rituals leaded by elders.

The example of Adrien’s journey at Takiwasi shows that the psychedelic ritual leads to the progressive emergence of experiences whose very content – as well as the interpretation they are subject to – are shaped by the explicit (discourses) and implicit (ritual actions) suggestions of the local epistemic authorities. The state of *hypersuggestibility* induced by psychedelics seems to play a key role here. As we could see, Adrien’s psychedelic experience is indeed progressively shaped by social interactions, and in particular by the suggestions of people perceived as epistemic authorities. The interactions with the ritual specialist, by describing part of Adrien’s experience of reality as the result of “parasitic” beings influence, is here a driving force. The infestation diagnosis, at first received with circumspection by Adrien, provokes indeed, in a dynamic evoking a form of “epistemic gaslighting”^25^ (Spear, 2019), a process of (self)-doubt and epistemic destabilization, eventually leading the participant to verify in an embodied fashion the belief system proposed by the ritual authority.

If the ritual experience is initially lived by the participant as a confusing one, the latter is then progressively shaped by the reverberating interplay of the participants’ accounts of his experiences and the comments of the ritual specialists, who gradually inscribe it in the local belief system. The “socialization of hallucinations” (Dupuis, 2021) dynamic can be clearly observed here: involvement in ritual and discursive interactions implies an education of attention and an associative learning that progressively shapes the inferences governing the apprehension of the psychedelic experience. The participant learns to spot the signs of culturally postulated entities’ presence first in the ritual context, then in his past and daily life. It is this concrete and tangible verification through the psychedelic ritual experience that finally leads Adrien to adhere to the local belief system. I claim consequently that the state of hypersuggestibility induced by psychedelics, by enhancing the susceptibility to the influence of external interpretations of one’s experiences, is a driving force in the socialization of hallucinations dynamic, and thus in the dynamics of transmission of beliefs, which, as we have seen, is here conditioned on the experiential verification of the object of belief.

## Conclusion: The Variable Destinies of Psychedelic Belief Transmission and Its Ethical Stakes

Drawing on an ethnographic case-study, I sought in this article to shed light on the vectors through which psychedelics supports persuasion, enculturation and belief transmission dynamics. Proposing to approach psychedelic rituals as *suggestibility techniques*, I claimed that the property of hallucinogens to produce a state of hypersuggestibility strongly supports these dynamics. It seems to be this remarkable property of psychedelic substances that has led the defenders of these practices to extol their “therapeutic” and “transformative” virtues, and their detractors to denounce them as vectors of “mental manipulation.”

Even if the psychedelic ritual strongly increases the suggestibility of participants, the psychedelic experience is far from inducing a sudden conversion and automatic persuasion, but seems rather lead most participants to an oscillating indetermination mobilizing the reflexivity and agentivity of the recipient. As illustrated by Adrien’s case, the psychedelic ritual leads participants to an oscillation between doubt and adherence which is paradoxically a driving force in the belief transmission dynamic. The dynamic of belief transmission relies indeed ultimately on the recipient’s efforts to verify the object of the belief through an *experiential verification* process. As we could see, this verification process relies on the socialization of hallucinations dynamic, which is sustained by the properties of psychedelics to enhance suggestibility.

The belief transmission based on the psychedelic ritual in Takiwasi, although frequently successful, is however, far from being automatic. The adherence aroused by the hallucinogenic experience appears rather as the fact of a progressive learning, carried out in a more or less fast and complete way according to the participants, and which will be moreover never accomplished by many of them. If the cases of “conversion” based on the hallucinogenic experience, exemplified here by Adrien, illustrate a very recurrent pattern in Takiwasi, this case should consequently not hide important inter-individual variations. The testimonies of the participants underline a great diversity in the importance given to the various elements composing the local belief system, which is far from being reduced to the pattern of infestation. In the course of my investigation in Takiwasi, I saw many of them being disappointed by the propositions of local epistemic authorities. As illustrated by the testimonies of participants stating during our interviews that they “will never come back to Takiwasi again,” the local belief system, and in particular the central place given to the motif of demonic possession, arouses in some of them a strong and definitive opposition. These observations highlight the continuing relevance of the proposals of the culture-and-personality school theorists (Kardiner, 1939; Piotrowski, 1946), which have shown that people always socialize on a selective basis: as they react according to various idiosyncratic factors, they participate actively in the dynamics of cultural evolution.

If by producing a community of experience and ensuring the transmission of a belief system, psychedelic rituals appears as powerful potential vectors of affiliation to the social group, the long-term maintenance of membership is also subject to great variability. In following up through interviews some of the seminar participants several years after their trip to the Amazon, I was struck by the great diversity in the credence they gave to the belief system they seemed to have adhered to during their stay at Takiwasi. The specificity of the French context, which is marked by the association of these practices with “sectarian drifting” by the public authorities, plays here a certain role. Back to France, the participants were often seized by doubt: had they had a spiritual and therapeutic experience, or had they been the object of “cult manipulation,” deluded by “hallucinogenic” substances? The long-term follow up of the participants’ journey shows that the fate of membership to what many of participants called “the Takiwasi spiritual family” is then relying on the frequency and maintenance of participation in the practices they experienced in Peru. In order to maintain this membership, participants must continue to participate in practices that are frowned upon in their own country. They have consequently to take the risk of breaking with the dominant representations of their social group of origin, and thus of being subjected to a socially stigmatizing identity: that of a follower of a “cult.” These elements are undoubtedly a driving force in the fact that many of them abandon, after some time, the belief system they adopted during their stay in Peru. Beyond the particular case of Takiwasi, these observations underline, as much as the power of the psychedelic experience in the dynamics of belief transmission, the central role of social interactions and institutions in the maintenance of adherence.

These few points show that although psychedelics strongly support belief transmission by enhancing suggestibility, this dynamic can’t be described adequately with the model of brainwashing, which has recently been associated with the use of psychedelics by some European public authorities and anti-cult associations. This controversed model, drawing on automatist dynamic inspired by behaviorist theories of learning, turns psychedelic users into automatons devoid of reflexivity and agentivity, passive carriers of cultural transmission which leads them mechanically to assent to a belief system. In contrast with this model, we could see here that the belief transmission dynamic is here extremely labile, woven of reflexivity and ambivalence. It seems finally that by denying the subject an agency of its own and by neglecting the active dynamism constitutive of cultural transmission, the brainwashing model prevents understanding belief transmission and religious conversion based on psychedelic experience. This dynamic can be best on psychedelics can be best understood in the light of social influence processes long studied by social psychology (Turner, 1991; Rashotte, 2007), which are here potentiated by the remarkable properties of these substances, as the hypersuggestibility state that they produce.

These observations highlight that despite its common usage in psychology and psychiatry, and the few attempts to apply this notion to anthropological analysis, the concept of suggestibility and its implications in the field of psychedelic studies deserves to be further explored^26^. While the ability of psychedelics to increase suggestibility (Leary, 1961; Grob and Dobkin de Rios, 1992; Carhart-Harris et al., 2015; Dupuis, 2021) and the weight of extra-pharmacological factors in the hallucinogenic experience (Hartogsohn, 2016) have been known for a long time, this feature raises indeed ethical concerns about the kind of influence therapists, shamans and other facilitators are having over their clients, even when therapy goes well. In a paper recently written with some colleagues (Timmermann et al., 2020), we argued that the specific features of the psychedelic experiences may act as a *double-edged sword*. While it may drive therapeutic benefits, the ability of psychedelics to induce hypersuggestibility as feelings of reverence and revelation might leads to problematic effects in the absence of ethical guidelines regulating their use, especially considering the blurry distinction between accepted and forced persuasion. In the context of the current globalization of psychedelics and while legal restrictions around these substances are now slowly relaxing in the countries making up the Global North, it now seems essential to think of ethical frameworks regulating the use of psychedelics, beyond the prohibitionist logic held in recent decades. In light of the remarkable properties of psychedelics highlighted in this article and the dynamics of “epistemic gaslighting” that they seem to facilitate, the question that should guide these considerations is not “does psychedelic brainwashing exists?,” but rather: to what degree can people be influenced through the use of psychedelics? What are the individual and relational factors that result in the strongest influence? Does the conditions of persuasion are ethical?^27^

## Data Availability Statement

The raw data supporting the conclusions of this article will be made available by the authors, without undue reservation.

## Ethics Statement

Ethical review and approval was not required for the study on human participants in accordance with the local legislation and institutional requirements. Written informed consent for participation was not required for this study in accordance with the national legislation and the institutional requirements.

## Author Contributions

The author confirms being the sole contributor of this work and has approved it for publication.

## Conflict of Interest

The author declares that the research was conducted in the absence of any commercial or financial relationships that could be construed as a potential conflict of interest.

## Publisher’s Note

All claims expressed in this article are solely those of the authors and do not necessarily represent those of their affiliated organizations, or those of the publisher, the editors and the reviewers. Any product that may be evaluated in this article, or claim that may be made by its manufacturer, is not guaranteed or endorsed by the publisher.

## Footnotes

1 The term ayahuasca refers both to a vine (*Banisteriopsis caapi*) and the brew of which it is the main ingredient. This psychotropic and emetic brew is traditionally used in the Western Amazon as part of indigenous and metis shamanic practices and has sparked growing interest among the international public in recent decades as part of the emergence of shamanic tourism and the development of Brazilian religious movements as *Santo Daime* and *União do Vegetal*.

2 These substances classically associated with the popular category of “psychedelic” or the medical category of “hallucinogen” produce very similar perceptual and cognitive changes and share a common neurophysiological mode of action, primarily mediated by agonism of serotonin receptors, and the serotonin 2A receptor subtype specifically (Nichols, 2004).

3 Among many others, we can mention the examples of the use of iboga in the Bwiti cult in Gabon (Fernandez, 2019), of datura among Chumash of California (Applegate, 1975) and Shangana-Tsonga of Mozambique (Johnston, 1972), of Pituri (*Duboisia hopwoodii*) among Australia Aborigines (Dobkin de Rios and Stachalek, 1999).

4 Victor Turner (1969) has coined this term to designate an experience of intense togetherness and shared condition that temporarily transcends social distinctions.

5 MIVILUDES (Mission interministérielle de vigilance et de lutte contre les dérives sectaires – in French – Interministerial Mission of Vigilance and Combat against Sectarian Abuses) is a French government agency created by presidential decree in 2002. It is responsible for observing and analyzing the phenomenon of so-called “cultish” groups, coordinating the governmental response, informing the public of the risks linked to sectarian abuses and facilitating the implementation of actions to assist victims.

6 The CIAOSN (Center for Information and Advice on Harmful Sectarian Organizations), which is the Belgian equivalent of MIVILUDES, emphasizes for instance in its 2010–2011 report to the Belgian Council of Ministers that the use of ayahuasca “leads to the adherence of the most vulnerable and suggestible subjects, to a rigidification of psychic processes, to an impoverishment and a narrowing of the affective life around this object, ayahuasca” (C.I.A.O.S.N, 2011, p. 21). France continues to play a leading role in the anti-cult movement in Europe and in its coordination, notably through the FECRIS (Fédération Européenne des Centres de Recherche et d’Information sur le Sectarisme) – (in French) European Federation of Centres of Research and Information on Sectarianism. This French non-profit anti-cult association founded in 1994, serves as an umbrella organization for groups which investigate the activities of groups considered cults in Europe.

7 Such as the Unification Church, the Divine Light Mission, The Way International, the International Society for Krishna Consciousness, and Church of Scientology.

8 Initially consisting of small and informal groups of distressed family members of individuals joining emerging religious movements, these groups gradually grew into larger organizations operating mainly as non-profit organizations centered on information, education and support of so-called “cult” members and their families. Attempts to create a national confederate organization, however, have always failed because of dissension over local autonomy and funding issues (Shupe et al., 2004).

9 Deprogramming practices included the abduction and forceful detention of a member of so-called “cult” by parents or anti-cult movement professional deprogrammer, and the subsequent application of pressure for the member to withdraw renounce their religious affiliation (LeMoult, 1978). The methods used in deprogramming have been described by many observers as being just as coercive than the methods the anti-cult movement claims were used by “cults” to indoctrinate their members (Young, 2012).

10 Academic work on thought reform programs has indeed shown that the Chinese Army did not have techniques capable of converting individuals to Communism against their wills (Schein, 1956; Robbins and Anthony, 1980). The techniques used in Chinese prison camps were generally quite ineffective, achieving only short-term and superficial behavioral changes, based on the plausible threat of physical coercion. Most scholars conclude that the few subjects who had been slightly more influenced by Communist indoctrination differed from the great majority of subjects because of personality features that existed prior to the indoctrination process, which predisposed them to respond favorably to totalitarian propaganda (Anthony and Robbins, 1994). Psychologist Robert Lifton, on whose work Singer had based his model, has finally denounced the applicability of his research on military indoctrination techniques to that of religious conversions and so-called “cultish brainwashing” (Shinn, 1993).

11 The origins of this federal case lays in a one million dollars fraud scheme Fishman conducted during the eighties. The FBI investigated the possibility of church involvement in the scheme, as 30% of the total amount of the fraud have been spent on Scientology materials and services. Fishman’s attorney used an insanity defense, offering false memory syndrome theorist Richard Ofshe and psychologist Margaret Singer as expert witnesses (Anthony, 2004).

12 Singer attempted to respond to these criticisms by stating, in the second edition of her book (2003), that brainwashing is involuntary not as “against the person’s will” but in the sense that the subject is unaware that there is an agenda to control or change the person and that this process is happening. However, this definition of brainwashing is so broad that it is difficult to see what distinguishes it from the persuasion procedures observed in numerous social areas (advertising, political campaigns, education, etc.) and, more generally, in any socialization dynamic.

13 *The Manchurian Candidate* is a novel of Richard Condon published in 1959, in which the soldier Raymond Shaw is brainwashed by communists after being captured during the Korean War. Back to civilian life in the United States, where he becomes an unwitting assassin in an international communist conspiracy to overthrow the United States government. This bestseller has been the subject of two successful film adaptations, in 1962 and 2004.

14 It should also be considered that as the main debates on the concept of brainwashing took place in the 1980s in the United States, most Europeans were not aware of these earlier debates, as the main documents were only available in the English language (Melton, 1999).

15 This law, so-called “About-Picard” law (law n°2001-504 of 12 June 2001, enshrined in article 223-15-2 of the French criminal code) punishes with 3 years imprisonment and a fine of €375,000 “the fraudulent abuse of the state of ignorance or weakness (.) of a person in a state of psychological or physical subjection resulting from the exercise of serious or repeated pressure or techniques likely to impair his or her judgment, to lead (.) that person to an act or abstention which is seriously prejudicial to him or her”.

16 In Germany, military research conducted by the Schutzstaffel (SS) and the Gestapo led to the testing of mescaline in order to improve coercive indoctrination techniques and interrogation tactics. In United States, a similar type of research undertaken by the Office of Strategic Services (OSS), and then continued by the CIA, used LSD for the same purpose. Common goals of these experiments was to create an effective method to convert enemy captives into deployable secrets agents, to train soldiers to remain strong in the face of captivity by enemy forces, or to develop effectual wartime propaganda. The results produced no evidence at all for the success of these researches, and researchers ultimately declared their work a complete failure (Anthony and Robbins, 1994).

17 However, this method will have limitations for so called “hard science” perspective, such as relying on a relatively small number of participants which does not allow for the development of meaningful statistical aggregates, on which, for example, most of the psychology studies are based.

18 *Malocas* are traditional Amazonian constructions, oval in shape with a conical roof.

19 Barks of a perfumed wood (*Bulnesia sarmientoi*) frequently used in the practices of the Peruvian *curanderismo*. Its use in Takiwasi is to purify and protect the ritual space.

20 Brown tobacco of a local variety (*Nicotiana rustica*).

21 *Agua florida* is a cologne used in the practices of Métis shamanism in South America as well as in Central America and the Caribbean as a purification, care and protection tool.

22 Medal given to Adrien by Jacques Mabit shortly before the first ayahuasca ritual. Engraved with the effigy of St. Benedict, the medal of St. Benedict represents the patron saint of monks attacking the devil, holding a crucifix in his right hand and pronouncing a conjuring formula reproduced on the medal. Medals and crosses of St. Benedict were used in Europe from the Middle Ages onward as talismans to protect people, animals, houses and fields from evil influences.

23 This distinction refers to dual-process models of cognition, contrasting intuitive processes (System 1) that are fast, automatic, and implicit on the one hand, and reflective processes (System 2) that are slow, deliberate, and explicit, on the other (Evans, 2008).

24 Suggestibility is understood here in its minimal definition, as a person’s propensity to respond positively to suggested communications, i.e., thinking and acting following the suggestions of others (Sidis, 1898).

25 Spear (2019) has emphasized the epistemic dimension of what popular psychology refers to as “gaslighting,” i.e., the process through which an agent lead another one to accept that its cognitive faculties, perceptions, judgments, and grasp of its own situation are deficients. This process, occurring in many interpersonal contexts involving trust or authority, has the consequence to build an authoritative relationship, by leading those who are subject to it to internalize a view of themselves that both neutralizes their ability to criticize authority figure and ensures their consent to the perspective proposed by this figure. As Robbins and Anthony (1980) had already observed, the practice of exorcism (which is based in Takiwasi on the diagnosis of infestation) is ironically reminiscent in this respect of the deprogramming practices carried out by the anti-cult movements in the 1970s. The notions of brainwashing and demonic entities are indeed both occult notions that lead to thinking of the individual as unconsciously manipulated by malevolent forces, and that legitimize practices of rehabilitation of behavior perceived as deviant.

26 A recent model of the brain action of psychedelics inspired by predictive coding, known as REBUS – which proposes that psychedelics render high-level beliefs and assumptions more plastic, and that this process is a driving force underlying their phenomenological effects and potential therapeutic outcomes -, may provide some inspiration for further investigations of the neurobiology of belief change processes under psychedelics and their link to increased suggestibility (Carhart-Harris and Friston, 2019; Timmermann et al., 2021).

27 An important criteria for determining the ethical character of psychedelic persuasion could be: is the subject aware that a process of persuasion and transmission of beliefs is here at work? Has this been clearly explained before participating in the psychedelic ritual and has the subject been able to give informed consent to this process?
